# Inverse Design of Molecular Qudits for Quantum Circuitry

**DOI:** 10.1021/acs.inorgchem.5c00298

**Published:** 2025-04-04

**Authors:** Edward Latham, Alice M. Bowen, Nicholas Cox, Nicholas F. Chilton

**Affiliations:** † Research School of Chemsitry, Sullivans Creek Rd, Acton, ACT 2601, Australia; ‡ Department of Chemistry, The University of Manchester, Oxford Road, Manchester M13 9PL, U.K.

## Abstract

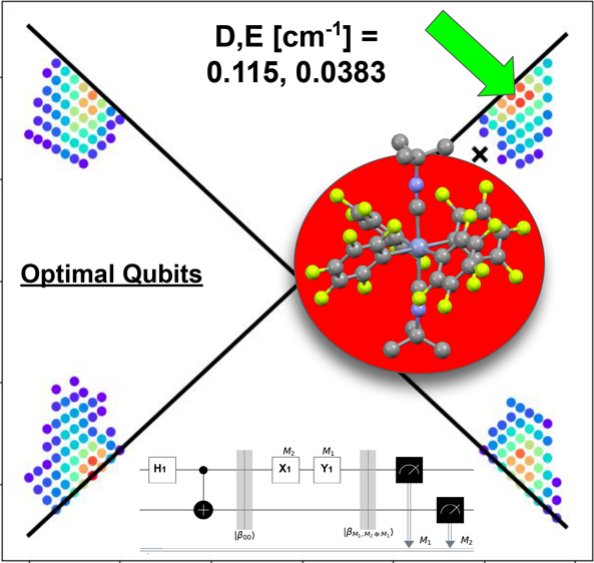

The development of molecular quantum bits (qubits) for quantum information processing is a lofty goal. While many contemporary works investigate their potential for error correction, fault-tolerance, memories, etc., there is still a lack of experimental examples of molecular multiqubit sequences. Herein, we perform a theoretical investigation of spin Hamiltonian parameter space to identify molecules that could be used to implement a 4-level superdense coding algorithm that has the least stringent requirements for experimental implementation. To do so, we analyze the zero-field splitting (ZFS) Hamiltonian of an *S* = 3/2 spin system to determine its effectiveness as a molecular qudit capable of performing the superdense coding circuit with X-band pulsed electron paramagnetic resonance (EPR), accounting for realistic constraints imposed by EPR spectrometers. For an *S* = 3/2 system, the optimal ZFS parameters are |*D*| ≈ 0.115 cm^–1^ and |*E*| ≈ −0.0383 cm^–1^ (|*E*/*D*| ≈ 0.33 approaching the rhombic limit of 1/3), with a field around 160 mT. Our findings highlight the need to maximize the rhombicity of the spin Hamiltonian for four-level molecular qudits.

## Abstract 2

Herein, we perform a theoretical investigation of spin Hamiltonian parameter space to identify molecules that could be used to implement a 4-level superdense coding algorithm that has the least stringent requirements for experimental implementation.

## 1 Introduction

First proposed explicitly by Deutsch in 1985,^1^ quantum information processing (QIP) is an ambitious application of quantum mechanics.^2^ QIP, and in particular, quantum computing, has the potential to vastly improve the performance of certain classical computational methods, such as prime number factorization, while completely transforming others, such as cryptography and database search algorithms.^3^ This will have enormous implications for the future of technology, both lowering the average energy consumption by developing more efficient methods and enabling previously impossible calculations.

QIP relies on performing logic operations on quantum bits (qubits), which are two-level quantum systems with certain properties^4^: they (1) are well-characterized and scalable, (2) are able to be initialized to a fiducial state, (3) have long coherence times, (4) facilitate a “universal” set of quantum gates, and (5) have a qubit-specific measurement capability. Although there are numerous different qubit implementations (e.g., nitrogen vacancy centers,^5^ phosphorus in silicon,^6^ superconducting transmons,^7^ trapped ions,^8^ photons^9^), which satisfy single-qubit logic operations (e.g., *X*, *Y,* and *Z* gates that rotate the state of a qubit^10,11^), a universal quantum computer requires qubits to interact with one another through multiqubit operations (e.g., CNOT, Swap, and Hadamard gates).^12^ Many platforms struggle with this step because the functionality gained by enabling multiqubit interactions can be a source of decoherence.^13^ While superconducting transmon qubits dominate the commercial QIP space due to their compatibility with existing semiconductor technology, exploring new qubit implementations that may natively support error correction strategies is desirable.^14^ Toward this goal, electron spins in molecules are a tantalizing possibility: they exist on nanoscale dimensions, permit chemical tunability, and can be designed with more than two degrees of freedom and as such act as qudits.^15,16^ The latter possibility can simplify the implementation of error correction schemes and enable custom Hamiltonian designs for specific problems. Indeed, nuclear spins in molecules can also contribute to the qudit space via hyperfine coupling, allowing multiple operation frequencies over a wide bandwidth. Despite many structural degrees of freedom leading to decoherence, molecular qubits have promising coherence times, ranging from microseconds at room temperature to milliseconds at low temperatures.^17^ However, the feasibility of using molecular hardware for quantum computations has not been extensively explored, save two nontrivial examples: the implementation of the Grover search algorithm^18^ and simulations of quantum tunnelling of magnetization and the transverse-field Ising model in a molecular qudit simulator.^19^ While work continues to improve the coherence properties of molecular qubits^20^ and plans are made for error correction codes,^21^ we should aim to demonstrate simple quantum circuits for multiple qubits as proof-of-concept of utility and generality.^22,23^

This approach also underscores the potential for custom molecules in constructing quantum circuits. By selecting molecules based on their Hamiltonian characteristics, one could build quantum molecular chips for quantum computing applications. Similar to how transistors can be configured as various logic gates, different molecules could be employed to perform specific qubit operations, thereby enhancing the functionality and efficiency of the quantum computing system as a whole.^24^ Thus, we advocate for the development of nonuniversal molecular qudits, tailored for specific tasks, leveraging the advantages of customizable Hamiltonians in molecular spin systems.

To address this challenge, herein we endeavor to find the requirements of a molecular qudit that can facilitate a simple yet nontrivial target algorithm requiring a qubit register of at least two qubits, that will showcase entanglement through superposition, manipulation of qubit states with various gates, and measurement of final states. For this purpose we have selected the superdense code,^25^ for which we lay out its translation to the electron paramagnetic resonance (EPR) paradigm, and determine the requirements of a molecular qudit that could facilitate this algorithm, accounting for both quantum mechanical and hardware limitations for manipulating the molecular eigenstates.

Our results present a method for determining optimal parameters for molecular qubits tailored to specific operations. We highlight the importance of maximizing rhombicity in the zero-field splitting (ZFS) tensor to enhance basis state mixing in conjunction with the Zeeman Hamiltonian (eq 1), thus facilitating more transitions in the Hilbert space than implied by selection rules in the canonical basis. Here,  is the spin vector operator (, , ), μ_B_ is the Bohr magneton, *g* is the electronic *g*-factor, and  is the magnetic field defined by its magnitude |*B*| and orientation θ_*B*_0__, ϕ_*B*_0__ with respect to the ZFS frame.
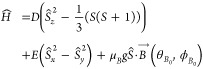
1

We find that for *S* = 3/2 molecules (such as octahedral chromium(III), octahedral manganese(IV) or tetrahedral cobalt(II)) operating with an X-band EPR spectrometer, spin Hamiltonian parameters *g* = 1.99, *D* = −0.115 cm^–1^ and *E* = −0.0383 cm^–1^ give Hamiltonians amenable to implementing the superdense coding algorithm with possible magnetic field orientations covering 17% of the sphere. This set of parameters implies a small, rhombic ZFS, which strongly implies a distorted octahedral chromium(III) complex is most suitable, for which we find several potential candidates in the literature.

## 2 Theory

### 2.1 Superdense Coding

**Figure 1:**
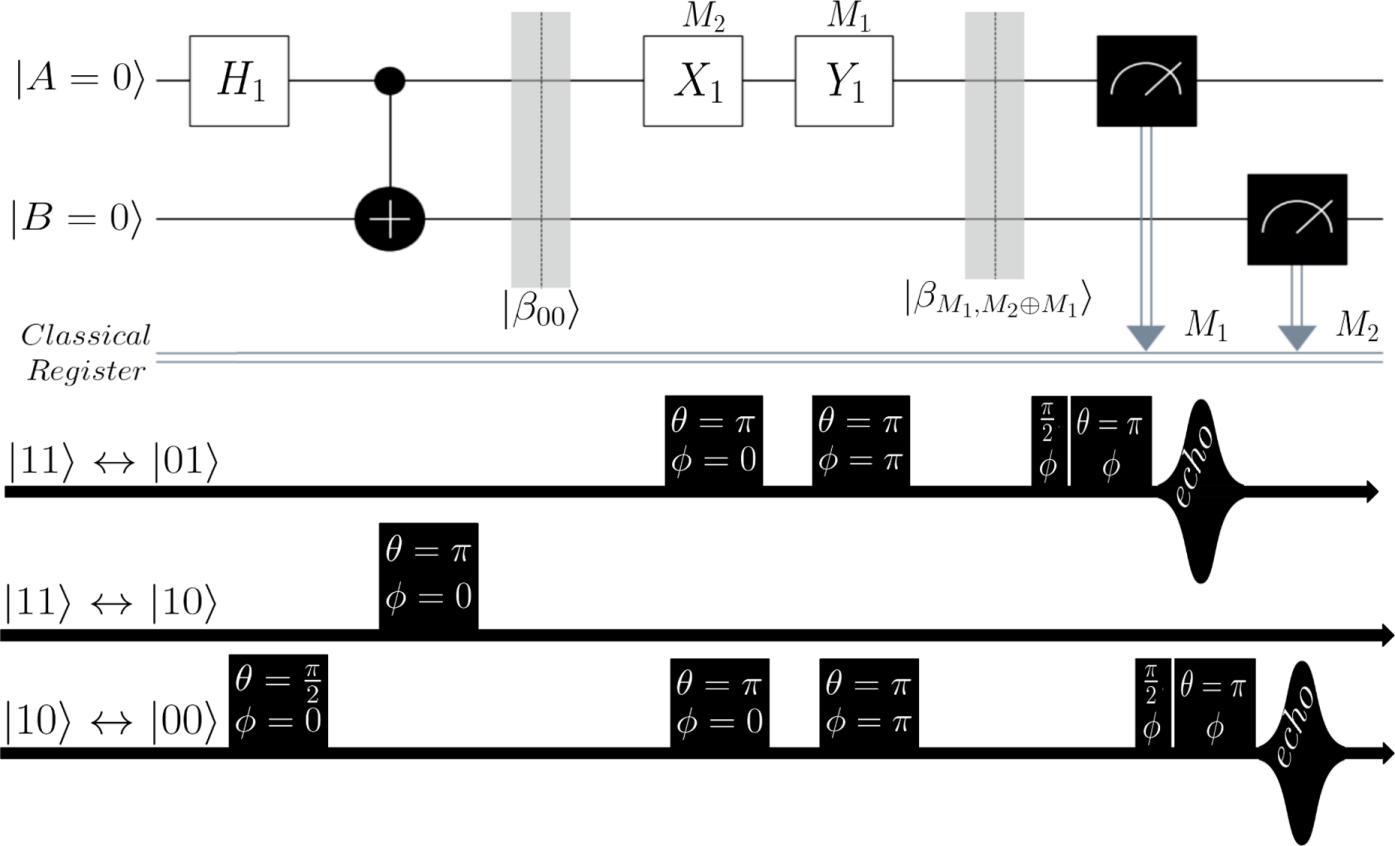
Superdense coding circuit diagram starts with 2 qubits, A and B, in state 0, then shows the preparation of an entangled state |β_00_⟩ by use of Hadamard and CNOT gates, followed by encoding of information through a series of quantum gates, *X* and *Y*, and the subsequent state measurements, *M*_1_, *M*_2_. Application or absence of *X* and *Y* gates are interpreted as the numerical values of *M*_1_ and *M*_2_ in the circuit diagram, having values 1 or 0, respectively. The circuit gates, *H*, *CNOT*, *X*, *Y*, map to the EPR pulse diagram below. Measurement of the final Bell state is performed using a Hahn echo. Note that the phase, ϕ, of the echo pulses changes to match the last gate used, ϕ_*X*_ ∨ ϕ_*Y*_ and that the resultant Bell state |β_*M*_1_,*M*_2_⊕*M*_1__⟩ uses ⊕ as the logic XOR gate because the traditional superdense coding circuit uses a *Z* gate which is not possible with conventional EPR spectrometers.

To effectively demonstrate the unique possibilities and functionality of molecular qudits, we have selected the two-qubit superdense coding circuit^26^ (Figure 1) which we propose to implement on a four-level molecular qudit. This circuit begins by entangling the qubits, manipulates their superposed states, and finally reads out the information. The four maximally entangled Bell states can be interconverted through the application of *X* and/or *Y* gates, and as such these gates indicated operating on the first qubit are either in the circuit, or not, which leads to four possible variations: *I*_1_, *I*_1_; *X*_1_, *I*_1_; *I*_1_, *Y*_1_; *X*_1_, *Y*_1_ (where *I* is the identity operator). Note that conventional EPR spectrometers cannot simply perform *Z*-gates, and as such we have altered the circuitry to use a *Y* gate instead of *Z*. To demonstrate unique identification of the final Bell states, which is crucial for showcasing the measurement capabilities of the molecular qudit, the circuit would be repeated with these four possible variations. The capability of manipulating multiple states with a single gate operation is where the circuit gets its name, where a single quantum gate can encode more information than possible with classical bits. For qudits with higher spin multiplicity, even greater information density can be achieved with fewer gates than classical computational methods.

Standard initialization schemes would start in the |00⟩ state and generate the Bell state  by applying a Hadamard gate to the first qubit followed by a controlled-NOT (CNOT) gate acting on the two-qubit system. The Hadamard gate *H*_1_ operating on the first qubit (note the subscript refers to ‘which’ logical qubit is being targeted for single-qubit operations) is
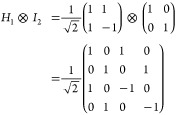
2and acting on the |00⟩ state results in
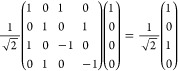
3

The CNOT gate is then defined as
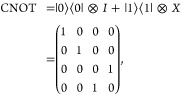
4and is then applied as
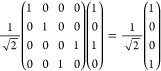
5which creates the first Bell state |β_00_⟩ as desired. Next, the manipulation gates are either *X*_1_ and/or *Y*_1_, which have matrix representations:
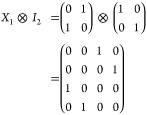
6
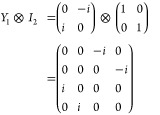
7

Application of *X*_1_ ⊗ *I*_2_ to |β_00_⟩ yields the |β_01_⟩ state:
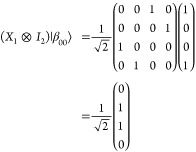
8

Similarly, the action of the *Y*_1_ gate additionally flips the phase and therefore gives access to the remaining Bell states by being applied on its own, or post the *X*_1_ gate. In summary:
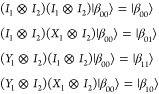
9

In our four-level quantum system, which will be operated in a nonzero magnetic field such that all degeneracies are removed, we assign the coupled qudit eigenstates for the *S* = 3/2 system to |00⟩, |01⟩, |10⟩, and |11⟩, in order of ascending energy. As such, finding the appropriate EPR pulse sequence to achieve the required gates is an exercise of mapping the EPR transition matrix onto the Kronecker product notation of the logical qubit basis states. To implement the superdense code in practice, we must translate the preceding gate operations into the EPR paradigm.

In pulsed EPR spectroscopy, we utilize resonant pulses to induce transitions between eigenstates of the total Hamiltonian (eq 1). Transitions between eigenstates correspond to a particular frequency, determined by the energy difference of those states (*h*ν_*ij*_ = *ℏ*ω_*ij*_ = Δ_*ij*_ = *E*_*j*_ – *E*_*i*_). By applying a resonant electromagnetic field at these frequencies, we can manipulate the coherences and populations of pairs of states on pairwise Bloch spheres. Using the conventional setup, the EPR microwave magnetic field *B*_1_ is perpendicular to the static external magnetic field *B*_0_. As such, in the canonical |*S*, *m*_*S*_⟩ basis, the transition matrix elements are dictated by the square modulus of the  operator (eq 10). While this operator seemingly only permits rotations between states differing by Δ*m*_*S*_ ± 1, in the eigenstate basis of the total Hamiltonian, the *m*_*S*_ basis states are sufficiently mixed such that there is nonzero probability for all transitions between our qudit basis states |00⟩, |01⟩, |10⟩, and |11⟩. Thus, it is important to understand the transition intensity of the microwave pulses, which directly specifies the required duration of each pulse to effect the chosen gate, ultimately dictating the length of the entire sequence. This has important consequences when considering decoherence rates and inhomogeneous broadening of the spin spectrum. In the context of a *S* = 3/2 system with ZFS, the transition intensity between arbitrary pairs can be increased by maximizing the rhombic component of the anisotropy, i.e., |*E*/*D*| → 1/3. As such, in large magnetic fields where the Zeeman term starts to dominate, the mixing from ZFS will be reduced, and arbitrary operations become harder.
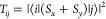
10

**Figure 2:**
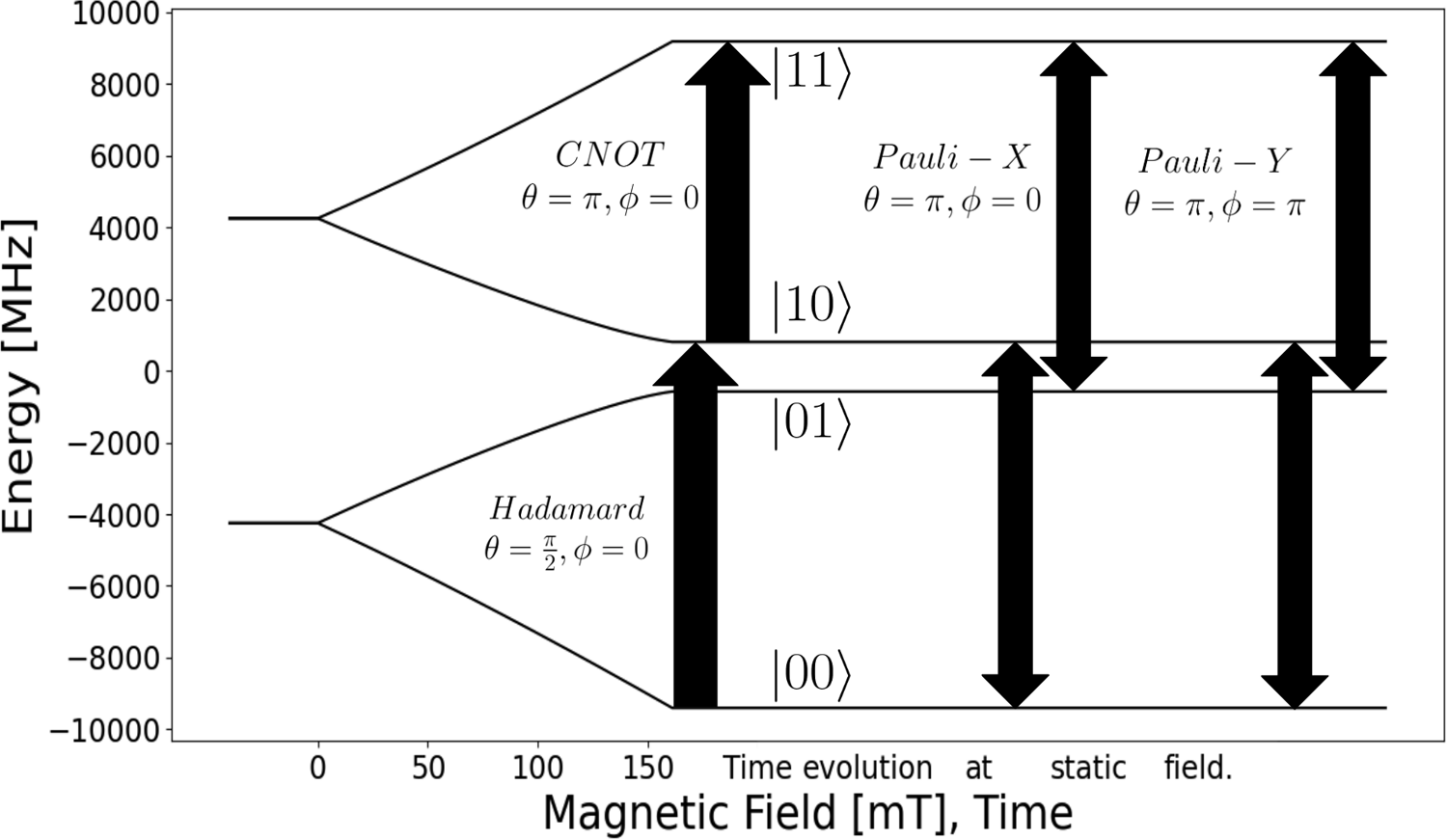
Energy spectrum for an *S* = 3/2 system under the influence of ZFS (for *D* = 0.13 cm^–1^ and *E* = −0.0346 cm^–1^) and Zeeman terms (for a magnetic field strength  mT at θ_*B*_0__ = 0.850, ϕ_*B*_0__ = 0.0798 rads), illustrating the bridge between energy states, EPR pulses, and quantum computing notation. The transitions between energy states are induced by EPR pulses, represented as *X* and *Y* gates in quantum computing. The *Y* gate is defined as an *X* gate with a phase modulation of π/2. The diagram depicts the initialization (Hadamard and CNOT) and manipulation (*X* and *Y*) of qudit states required for the superdense coding circuit in a 4 level qudit system.

In the context of a spin *S* = 3/2 system initialized in the |00⟩ eigenstate, the Bell state |β_00_⟩ can be obtained by a π/2 pulse, rotating the initial state around the *x*-axis defining the relative phases of the pulse sequence into the *x*–*y* plane of the Bloch sphere implied by the |00⟩ and |10⟩ pair; that is, a π/2 pulse on resonance with *h*ν = *E*_10_ – *E*_00_. This pulse rotates the magnetization vector around the *x*-axis to equalize the probability of being in either the |00⟩ or |10⟩ states of the coupled qudit (Figure 2, ‘Hadamard’ arrow). The CNOT gate can then be applied as a π pulse between the |10⟩ and |11⟩ eigenstates on resonance with *h*ν = *E*_11_ – *E*_01_, in phase with the first pulse (Figure 2, ‘CNOT’ arrow), taking care to have a sufficiently narrow bandwidth as to not also drive the |00⟩ to |01⟩ transition.

The Pauli-*X* gate is analogous to a classical bit flip, i.e., flip the first bit of our two entangled qubits in the Bell state in phase with the first pulse. Getting this pulse correct will be difficult because the action of this gate has to account for both |00⟩ to |10⟩ and |01⟩ to |11⟩ transitions in the qudit basis states (Figure 2, ‘Pauli-*X*’ arrow), as indicated by the off-diagonal elements in positions (3,1), (4,2) and (1,3), (2,4) (eq 6). This would require a careful choice of central pulse frequency, shape, and duration to drive this gate with sufficient fidelity.

The *Y*-gate drives the same transitions and requires the same shaped π pulse as for the *X*-gate but rotates the magnetization around the *y* axis to achieve a state that is π/2 out of phase compared to the *X*-gate.

In summary, to implement the quantum gates:

- Initialisation of Bell state: apply a resonant π/2 pulse that creates a superposition of eigenstates |00⟩ and |11⟩.
- Pauli *X*-Gate: apply a shaped π pulse near the resonant frequency for the two transitions corresponding to the first logical qubit, effectively coupling states |00⟩ to |10⟩ and |01⟩ to |11⟩ around the *x*-axis.
- Pauli-*Y* Gate: apply a shaped π pulse (same frequency, shape and duration as for X-gate), but with a π/2 phase shift to drive rotation around the *y*-axis to induce a phase shift in the magnetization vector.

These pulses will need to be carefully shaped to ensure that they drive the transitions selectively and achieve their desired rotations through π/2 or π pulses. Ultimately, we require pulse durations that are suitably shorter than the coherence time of the quantum states. The final stage involves measurement of the Bell states through on-resonance spin echo techniques and quadrature detection. Similar to a standard Hahn-echo sequence, after the last gate of the circuit a  pulse on resonance with one of the two pulses involved in each of the *X* and/or *Y* gates (and with the same phase) causes one of the components of the Bell state to rotate into the transverse plane of the corresponding Bloch sphere, which is then allowed to evolve for for time τ, before a refocusing π pulse inverts the qudit around the last rotated axis (*x* or *y*), reversing phase errors accumulated during the dephasing period. The echo signal is detected at time τ after the refocusing pulse, where the quadrature components (in-phase, I, and quadrature-phase, Q) of the echo signal, along with the negative or positive sign of the echo, provide the necessary information to determine the qudit state. There should be redundant information in each component of the Bell state, meaning that the echo obtained from using either the |10⟩⇔|00⟩ or |11⟩⇔|01⟩ frequency channel should be identical in quadrature; however, to prove the fidelity of the experiment, both channels should be measured in repeat experiments.

To maximize the fidelity of detection, deviations in the timing and amplitude of the π refocusing pulse must be minimized by accurate calibration of the optimal pulse. Although the spin echo technique corrects for some environmental interactions (e.g., inhomogeneous broadening), residual decoherence can still impact measurement fidelity. Enhancing noise filtering and shielding capabilities of the detector will also be required to achieve optimal fidelity. The final requirement for a practical implementation is state preparation, which is a challenge for molecular qubits. Recently, the pseudopure state method, which initializes the molecule with three pulses to average the population in all but the ground state, has been employed successfully for a molecular qubit.^19^ This would add to the length of the entire pulse sequence by an upper bound of 150 ns.

## 3 Results and Discussion

### 3.1 Instrumental Limitations

With a plan for implementation of the superdense code in the EPR paradigm, we must find an appropriate spin Hamiltonian parameter set that will enable the pulse sequence in practice, subject to instrumental and molecular constraints. Based on our need to implement resonant pulses between different states to effect π or  rotations, we need to know the relative duration of each pulse. The tipping angle θ of a microwave pulse is linearly proportional to the magnitude of the perturbing microwave field |*B*_1_|, the pulse duration *t*_*p*_, and the transition intensity between states |*i*⟩ and |*j*⟩, *T*_*ij*_ (eq 10). Intrinsically weaker transitions will require longer pulses to achieve the same tipping angle compared to more allowed transitions. To optimize successful operations within the coherence time of the qudit, we must find a Hamiltonian that supports transition intensities that are not drastically dissimilar throughout the manifold. To limit our search space, we set a requirement that all transition intensities are at least 10% that of an ideal basis state transition in the *S* = 3/2 manifold |⟨*m*_*s*_ = +3/2|(*S*_*x*_ + *S*_*y*_)|*m*_*s*_ = +1/2⟩|^2^ = 0.75, giving a lower limit of *T*_*ij*_ > 0.075. Note that the turning angle is proportional to the square root of the CW EPR transition intensity .
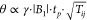
11

Our next consideration is to the practical implementation of pulses. Conventional EPR spectrometers are designed around single fixed frequencies, which, in principle, are tuned with a bandwidth of ca. 1 GHz. However, it is important to note that the power profile across the bandwidth is not uniform. Furthermore, the signal generator, usually an arbitrary waveform generator (AWG) and amplifiers have their own nonlinearities and bandwidths to consider. Finally, we must also recognize that even with a perfect square microwave pulse, the Fourier transform is a sinc function with a finite bandwidth of frequency content Δν that is proportional to the length of the pulse . Hence, a shorter pulse results in a larger bandwidth, allowing for more efficient excitation of a range of transitions, but also making the system prone to unwanted excitations. Considering all of these points, we set another requirement of at least 200 MHz differences between the nearest uniquely driven transitions to prevent leakage to undesired states during the sequence. This corresponds to pulse durations of <50 ns, which balances molecular qubit/qudit coherence times (accounting for successive pulses) and required differences in frequency to avoid driving unintended transitions but competes with sufficiently fast pulse times to maintain the sum of pulse lengths being less than the coherence time of the spin system. Such a pulse duration is short enough to ensure that multiple pulses can be issued within a *T*_2_ of 1 μs with high fidelity.

To summarize, the design and implementation of superdense coding circuitry on a four-level qudit are governed by the following critical quantities that ensure effective qudit manipulation and high-fidelity operations.

- Transition intensities greater than 0.075.
- Frequency range of all transitions between 8 and 10 GHz.
- Pulse duration 10 < *t*_*p*_ < 50 ns.
- Indicating energy eigenstates separated by >200 MHz.

Collectively, these quantities define the operational limits of our search for the ideal spin Hamiltonian and hence molecule, highlighting the balance between theoretical ideals and practical constraints in molecular QIP.

### 3.2 Finding Qudits

**Figure 3:**
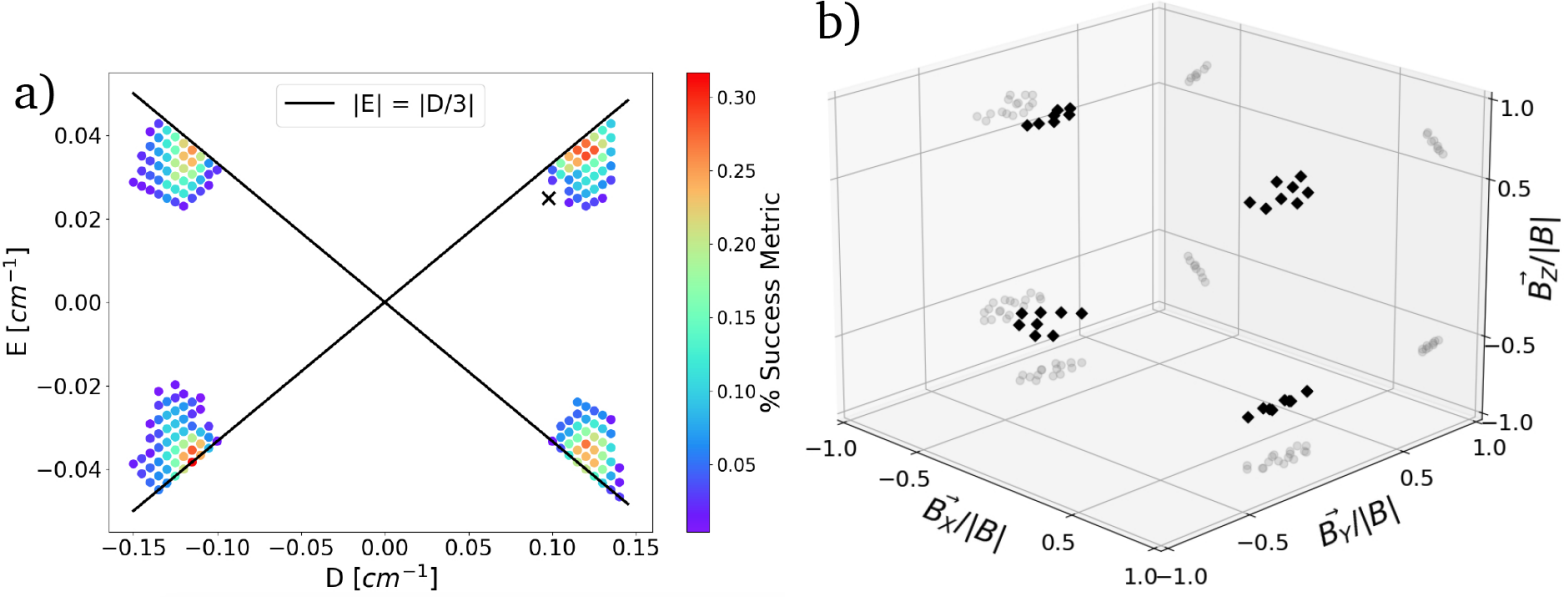
(a) *D* and *E* values sampled between ±0.2 cm^–1^ for the *S* = 3/2 Hamiltonian at magnetic field strengths 0–500 mT over 200 orientations. At each point in the 5-dimensional solution space, the transition frequencies and intensities are calculated and checked against the required parameters to return a pass/fail. The success metric is the percentage of successes for a given choice of *D* and *E*. We annotate with a cross *D* and *E* values for the molecule trans-[Cr(C_6_F_5_)_4_(CN^t^Bu)_2_]^−^^27^ that has very near to the optimal ZFS parameters. (b) Magnetic field vectors for *D* = −0.115 cm^–1^ and *E* = −0.0383 cm^–1^, for which the criteria (List 3.1) for superdense coding is satisfied. Rotations of the magnetic field vector are defined in the ZFS frame.

In order to find a set of conditions that fit the operational limits given above, we iterate through various ZFS parameters, magnetic field strengths, and orientations to generate potential spin Hamiltonians (eq 1). For each potential spin Hamiltonian, we determine the eigenstates by matrix diagonalization and, hence determine all transition frequencies and intensities. The solution space for the ZFS was defined within 0.2 > *D* > −0.2 cm^–1^, with a step of 0.05 cm^–1^, and −|*D*|/3 > *E* > |*D*|/3 in steps of |*D*|/100 cm^–1^. For each pair of *D* and *E* values, the magnetic field was varied within 0 > *B* ≥ 500 mT in 5 mT increments (100 points), and its orientation was sampled using a golden spiral distribution of 200 orientations (θ_*B*_0__, ϕ_*B*_0__) to cover equal surface space on a unit sphere.^28^ In the two-dimensional parameter space spanned by this range of *D* and *E*, a successful parametrization is one that yields eigenstates that obey the defined criteria above; we present results of the search as the fraction of magnetic field vectors (strengths and orientations) for a given choice of *D* and *E* that are successful (Figure 3a). Most success is found in regions where the ZFS approaches the rhombic limit |*E*/*D*| = 1/3. We can then determine the ideal magnetic field strength and orientation by selecting a parameter set, for example, *D* = −0.115 cm^–1^, *E* = −0.0383 cm^–1^, |*E*/*D*| = 0.333, and examining successful magnetic fields on a more dense grid (Figure 3b). These results show that our protocol requires significant orientation selectivity, where only 17% of magnetic field vectors on the sphere are successful. While the experiment is invariant to the molecule being ‘upside down,’ as anticipated through inversion symmetry of the magnetic field, the choice of the direction of the applied magnetic field vector in the *x*–*y* plane is dictated by the relative sign of *E* so that mixing between basis functions ensures sufficient transition intensity. Furthermore, if the spectrometer frequency was chosen differently, say Q-band at 34 GHz rather than X-band at 9.4 GHz, then the magnetic field strength and magnitude of the ZFS would necessarily need to increase to maintain 3*D* ≈ *h*ν at resonance, which maximizes mixing of the basis states in the molecular Hamiltonian. Then, the same selection criteria near the |*E*/*D*| rhombic limit and the same orientation selectivity would continue to apply.

**Figure 4:**
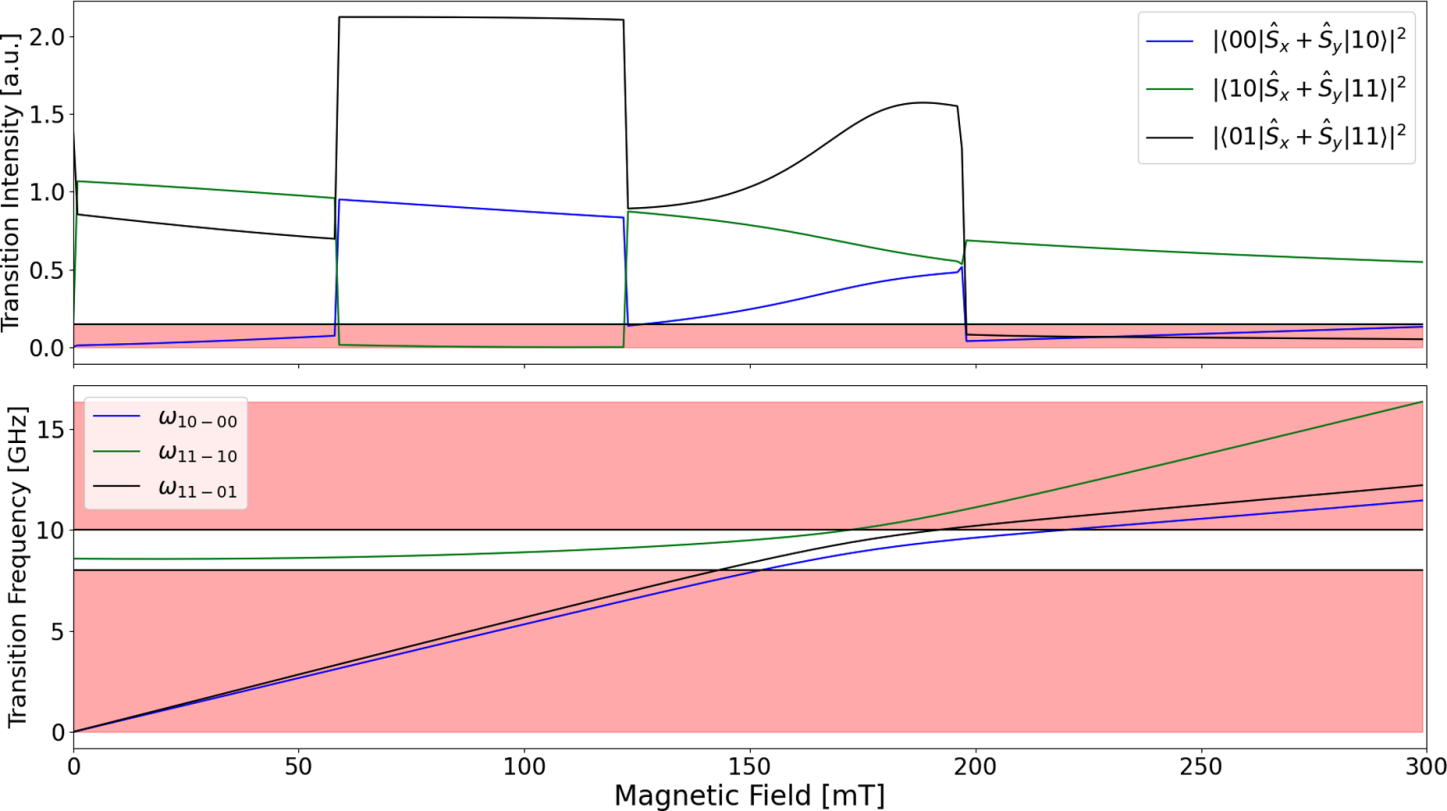
Transition intensity and transition frequency of the ideal Hamiltonian that satisfies the restrictions, identified in list 3.1, to enable superdense coding to be conducted with X-band EPR. The Hamiltonian has values *D* = 0.13 cm^–1^, *E* = −0.0346 cm^–1^, with magnetic field  and θ_*B*_0__ = 0.850, ϕ_*B*_0__ = 0.0798 rads with respect to the ZFS frame to demonstrate the finite specific conditions necessary for the pulse sequence to be possible, i.e.,  as shown. Red areas indicate where conditions are not met.

**Figure 5:**
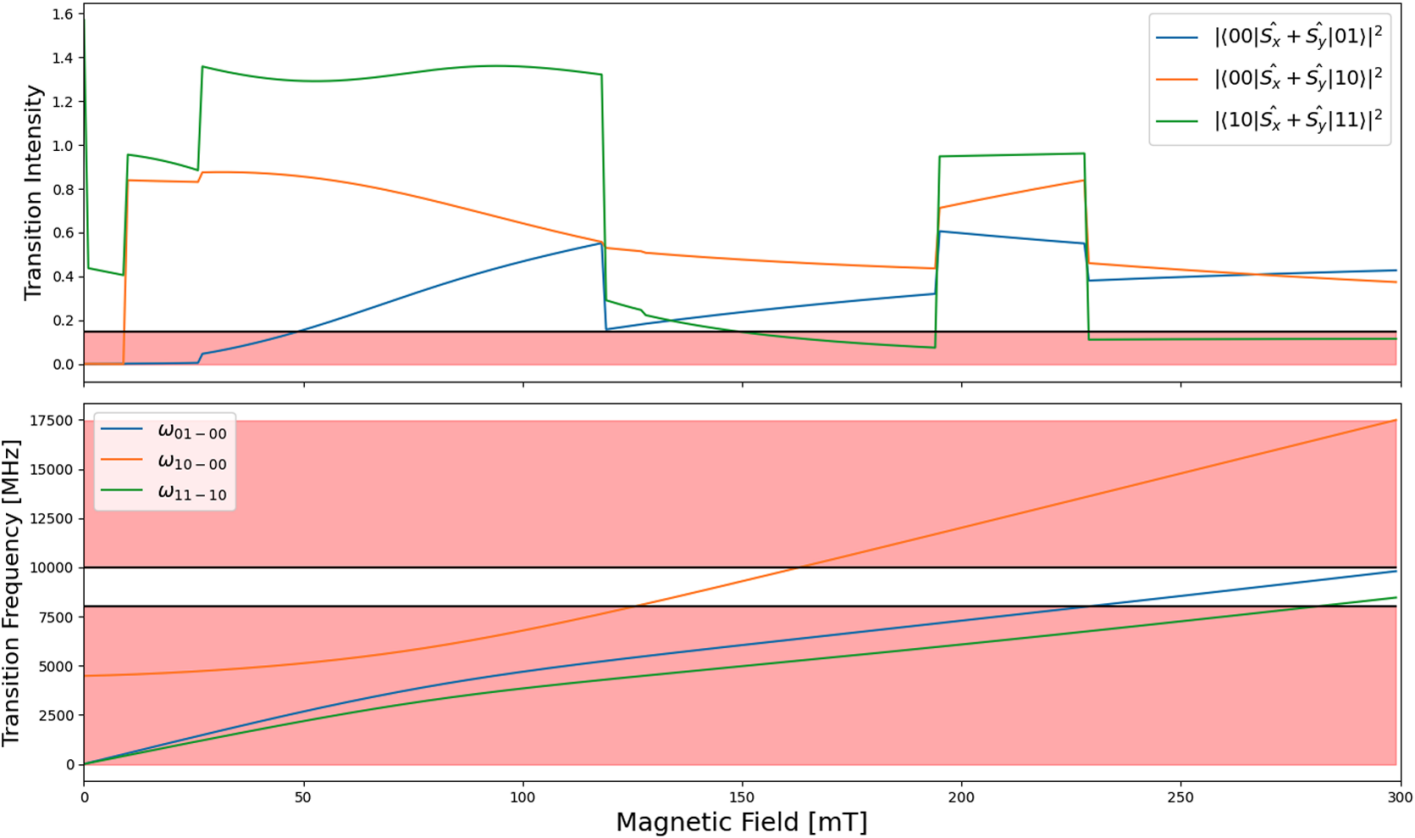
Transition intensity and transition frequency of trans-[Cr(C_6_F_5_)_4_(CN^t^Bu)_2_][NBu_4_] after aging for one year with *D* = 0.0977 cm^–1^, *E* = 0.0250 cm^–1^ with a magnetic field orientation of θ_*B*_0__ = 0.850, ϕ_*B*_0__ = 0.0798 rads with respect to the ZFS frame, to demonstrate the finite specific conditions necessary for the pulse sequence to be possible. Red areas indicate where conditions are not met.

**Figure 6:**
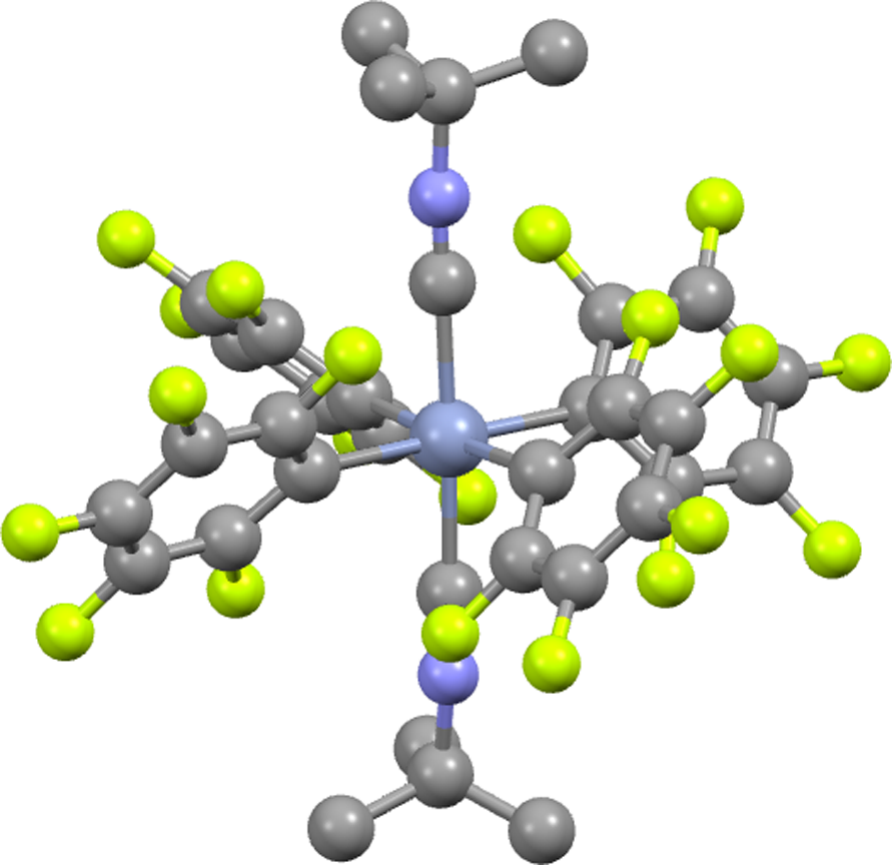
Rendering of the trans-[Cr(C_6_F_5_)_4_(CN^t^Bu)_2_]^−1^ anion.^27^ Steel blue = Cr, blue = N, gray = C, yellow = F, H atoms omitted for clarity.

Now that we have identified the parameters of an ideal *S* = 3/2 molecule, we have searched the literature for potential candidates to embody this qudit. Given that we would like the Zeeman and ZFS Hamiltonians to be of similar magnitude to maximize state mixing, we seek relatively weak ZFS on the order of ≈1 cm^–1^, similar to X-band microwave energy of *h*ν ≈ 0.33 cm^–1^. This immediately suggests a ^4^A ground term, which indicates octahedral chromium(III) with a 3d^3^ (t_2g_^3^) ground state. The most similar *E*/*D* ratio to our ideal scenario that we found in the literature is the one-year-aged sample of trans-[Cr(C_6_F_5_)_4_(CN^t^Bu)_2_][NBu_4_] (Figures 5 and 6).^27^ This compound has *D* = 0.0977 cm^–1^ and *E* = 0.025 cm^–1^ with *E*/*D* = 0.27 (Figure 3a, cross). Unfortunately, the code finds no successful magnetic fields when searching the solution space given these *D* and *E* values because they are too small to generate the transition frequencies that fall within our defined criteria. However, either a small tuning of the ZFS (either by a subtle change in chemical formulation or by extrinsic manipulation, for example, isolating this molecule in a different host or in the amorphous phase) or by using a spectrometer with a slightly larger bandwidth may allow the superdense coding algorithm to be implemented using this molecule. Indeed, there are several octahedral chromium(III) examples with a range of ZFS parameters that straddle the ideal parameter set (Table 1), indicating that a molecule could be constructed to have the desired parameter values (Figure 4). Another avenue for finding potential viable molecular qudits could be using ab initio calculations; this will be the topic of an upcoming work.

**Table 1:** Potential Molecules for Hosting the Superdense Coding Algorithm^a^

species	*D* [cm^–1^]	*E* [cm^–1^]	|*E*/*D*|	rejection reason
Cr^3+^ ions in [YGa_3_(BO_3_)_4_]^29^	–0.465	–0.013	0.028	|*D*| > 0.18
[Cr(ox)_2_(phen)][Cr(ox)(phen)_2_]·3H_2_O^30^	0.611	0.1572	0.26	|*D*| > 0.18
K[Cr(ox)_2_(bpy)]·5H_2_O^30^	0.572	0.114	0.20	|*D*| > 0.18
[Cr(phen)_2_(ox)]Cl·4H_2_O^30^	0.398	0.124	0.31	|*D*| > 0.18
Cr^3+^ in [Ga(acac)_3_]^31^	0.593	0.03	0.051	|*D*| > 0.18
trans-[Cr(C_6_F_5_)_4_(CN^t^Bu)_2_][NBu_4_]^27^	0.0747	0.0112	0.15^b^	|*D*| < 0.9
trans-[Cr(C_6_F_5_)_4_(CN^t^Bu)_2_][NBu_4_] aged 1 year^27^	0.0977	0.0250	0.27^b^	*E*/*D* is not negative

a ox = oxalate, phen = 1,10-phenanthroline, bpy = 2,2′-bipyridine, acac = acetylacetonate, ^t^Bu = C(CH_3_)_3_.

b Molecules were recorded with |*D*| and |*E*| and as such their rhombicity *E*/*D* may be negative and therefore closer to ideal values.

### 3.3 Implementation

**Figure 7:**
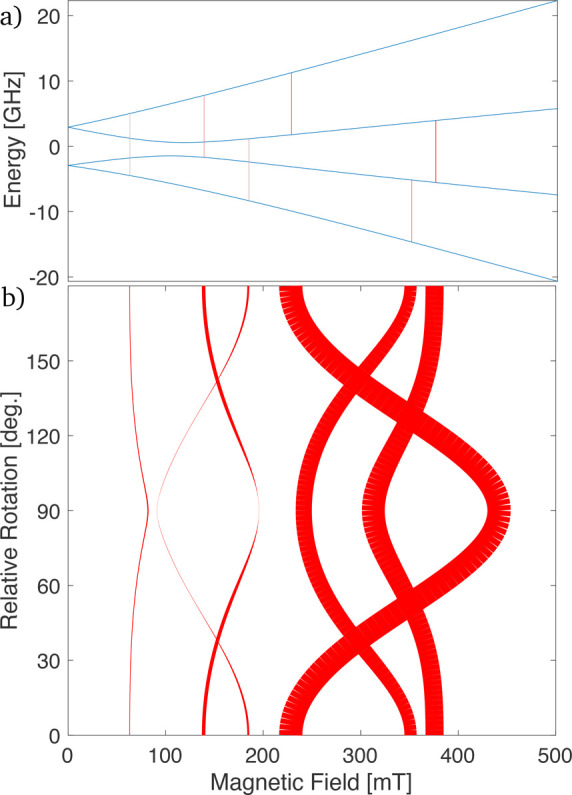
(a) Simulated continuous wave EPR spectrum of trans-[Cr(C_6_F_5_)_4_(CN^t^Bu)_2_][NBu_4_] at 9.5 GHz, where the magnetic field is applied at 43° to the principal axis of the ZFS tensor, showing the three transitions required for superdense coding between 100 and 300 mT. (b) Roadmap of the 9.5 GHz EPR transitions as a function of field orientation; the field starts at 43° to the principal axis and then is rotated 180 about the *x*-axis of the ZFS tensor. The thickness of the line represents the relative transition intensity.

Implementation of the super dense coding algorithm will be difficult due to the specific orientation required (Figure 3b). However, by using a single crystal doped with ≤1% Cr(III) in a diamagnetic host (e.g., Ga(III)), the orientation selection should be possible; the predicted single-crystal EPR roadmap for trans-[Cr(C_6_F_5_)_4_(CN^t^Bu)_2_][NBu_4_] shows the required transitions clustered at low-field (Figure 7).

## 4 Conclusions

In this study, we have explored the parameter space of *S* = 3/2 molecules to identify those most suitable for superdense coding, given realistic requirements imposed by current EPR technology. Our analysis focused on the ZFS Hamiltonian of octahedral chromium(III), highlighting several key insights and underscoring the importance of maximizing the rhombicity of the spin Hamiltonian for four-level molecular qudits. By publishing these results, we hope to inspire the inorganic chemistry community to find more suitable molecular systems that fulfill the required criteria. We find optimal ZFS parameters of *D* = 0.13 cm^–1^ and *E* = −0.0346 cm^–1^ for operation at X-band microwave frequencies. We find an exemplar molecule in the literature that possesses nearly exactly the required ZFS parameters, indicating that the present theoretical proposal could indeed be implemented with only minor adaptations. Future research should focus on techniques to either tune molecular ZFS or improve bandwidth in EPR spectrometers that would further improve the viability of superdense coding protocols executed on molecular qudits.

## Data Availability

The data and code that support the findings of this study are openly available on Github at https://github.com/EdL47ANU/InvMolQudits.
